# A randomized clinical trial evaluating the effects of administration of acidogenic boluses at dry-off on rumination and activity behavior in the 14 subsequent days

**DOI:** 10.3168/jdsc.2022-0366

**Published:** 2023-04-20

**Authors:** C.C. Florentino, E. Shepley, M. Ruch, M. Mahmoud, L. Tikofsky, W.A. Knauer, G. Cramer, S.M. Godden, L.S. Caixeta

**Affiliations:** 1 Department of Veterinary Population Medicine, University of Minnesota, Falcon Heights, MN 55108; 2 Department of Animal Medicine, Faculty of Veterinary Medicine, Beni-Suef University, Beni-Suef, Egypt 62511; 3 Boehringer Ingelheim Animal Health USA Inc., Duluth, GA 30029

## Abstract

Elevated milk production at dry-off can lead to increased udder pressure and, in turn, increased stress due to pain and discomfort, affecting natural behaviors. Administering acidogenic boluses at dry-off acts by inducing temporary and mild decreases in blood pH. This decreases dry matter intake, reduces milk yield, and increases cow comfort by lessening udder pressure. The objective of this study was to assess the effect of oral administration of acidogenic boluses at dry-off on total daily activity (TDA) and total daily rumination (TDR) behaviors in the first 2 wk of the dry period. This randomized clinical trial was conducted on a single farm and cows were randomly assigned to either treatment (TRT; n = 30) or control (CON; n = 34). The TRT group received 2 acidogenic boluses at dry-off and the CON group received no intervention. All cows received dry-cow therapy (intramammary antibiotic and internal teat sealant). The TDA and TDR data from 7 d before to 14 d after dry-off were measured using ear-mounted activity monitors. Analyses were performed using linear mixed-effects models with repeated measures. We observed a similar TDA in both groups throughout the study follow-up period. Overall, cows in the TRT group spent 17 min/d less time active than cows in the CON group in the first 2 wk after dry-off with the greatest difference observed on the second day of the dry period (TRT = 395 min/d; 95% CI: 370 to 420 vs. CON = 428 min/d; 95% CI: 404 to 451). The TRT group had lower TDR in the first 24 h after bolus administration (TRT = 437 min/d; 95% CI: 414 to 461 vs. CON = 488 min/d; 95% CI: 466 to 510) when compared with the CON group, but no differences were observed when comparing both groups in the 13 subsequent days. Our results indicate that administering acidogenic boluses at dry-off slightly decreased TDA during the first 2 wk of the dry period and decreased TDR on the first day after administration.

## Highlights

• Cows receiving acidogenic boluses were less active during the first week post dry-off.
• Cows receiving acidogenic bolus had the most pronounced decrease in 24 hours after dry-off.
• Acidogenic boluses had minimal biological effects for dairy cows.

## Graphical Abstract

**Figure:**
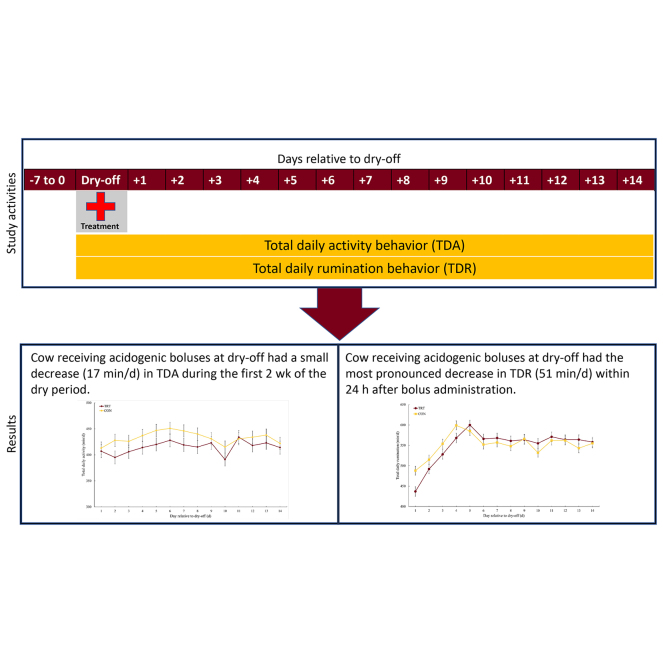
**Summary:** The effects of the administration of acidogenic bolus at dry-off on total daily activity (TDA) and total daily rumination (TDR) behaviors during the 2 weeks following dry-off were investigated in this randomized clinical trial. Cows in the treatment group (TRT) received 2 oral acidogenic boluses at dry-off, whereas cows in the control group (CON) did not receive any intervention. The TRT cows were less active in the first 2 weeks post dry-off with a decreased TDR observed only in the first day after acidogenic bolus administration. The results indicate that the administration of acidogenic boluses at dry-off should be considered as an alternative management strategy to increase cow comfort in the first 2 weeks after dry-off.

The dry period, defined as the period of nonlactation before parturition, provides a respite for the cow, allowing the secretory tissue of the mammary glands to regenerate to maximize milk yield in the next lactation (Weber et al., 2015). Elevated milk production at dry-off, however, can have negative impacts on cow health and welfare during the dry period. Cows producing more than 12.5 kg/d of milk at dry-off are at a greater risk of having IMI at calving, potentially because of milk leakage and delayed formation of the keratin plug after dry-off (Rajala-Schultz et al., 2005). Although cows are not milked after dry-off, milk is still being synthesized during the early dry period, leading to an increase in udder pressure, which can be a source of discomfort and even pain (Bertulat et al., 2013). Hence, changes to cow lying behavior may reflect increased udder pressure, as standing could provide a means of avoiding udder discomfort (Chapinal et al., 2014). For instance, daily lying time in the days following dry-off was reported to be negatively correlated with milk production at dry-off, with high-producing cows spending the least amount of time lying down (Rajala-Schultz et al., 2018). The authors hypothesized that this observation was associated with a greater increase in udder pressure after dry-off in high-producing cows when compared with low-producing cows.

Toward the goal of a successful dry period, different methods have been studied to decrease milk yield at dry-off and prevent milk leakage and new IMI during the dry period. Some of the strategies that have been investigated include a decrease in the quality or quantity of the feed offered to cows in the weeks before dry-off (Odensten et al., 2005; Ollier et al., 2014), decreased milking frequency and gradual cessation of milking (Tucker et al., 2009), and the use of pharmacological agents (e.g., casein hydrolysate and prolactin-release inhibitors; Ponchon et al., 2014; Bach et al., 2015). The use of acidogenic boluses in late-lactation cows has recently been demonstrated to decrease milk yield of high-producing dairy cows, resulting in lower udder pressure, particularly in the first 2 d after dry-off (Maynou et al., 2018).

The administration of acidogenic boluses containing ammonium chloride, a strong acidifying agent, leads to a temporary state of metabolic acidosis (Maynou et al., 2018). Severe metabolic acidosis has been associated with decreased rumination time (DeVries et al., 2009), whereas mild metabolic acidosis, such as that observed when feeding diets with negative dietary cation-anion differences (Goff et al., 2020), has not. While urine pH measured after the administration of acidogenic boluses is consistent with only a mild metabolic acidosis (Maynou et al., 2018), the effect of acidogenic boluses on rumination activity has not been investigated.

The administration of acidogenic boluses at dry-off is a promising additional strategy for the management of cows at dry-off, as it can reduce milk production and potentially improve cow welfare without requiring alterations to the management of feeding or milking strategies. Thus, the objective of this randomized clinical trial was to evaluate the effects of the administration of acidogenic boluses to dairy cows at dry-off on (1) total daily activity (**TDA**) and (2) total daily rumination (**TDR**) in the first 14 d of the dry period. We hypothesized that the administration of the acidogenic boluses at dry-off would decrease TDA, but not TDR, in the first few days after administration.

All interventions and procedures in this study were approved by the University of Minnesota Institutional Animal Care and Use Committee (2012–38706A). The study was conducted from April to September of 2021.

In total, 64 Jersey-Holstein cross cows were enrolled from a single commercial dairy farm in Minnesota. Pregnant cows (218 ± 13 d carrying calf) were randomly assigned to 1 of 2 groups at dry-off. Treatment allocation followed a random list of number, blocked by week, generated using Microsoft Excel 2016 (Microsoft Corporation). Farm personnel were blinded to study groups, but the research team was not. Cows in the treatment group (**TRT**; n = 30) received 2 acidogenic boluses orally (Bovikalc Dry; Boehringer Ingelheim Animal Health), whereas cows in the control group (**CON**; n = 34) did not receive any boluses at dry-off. All cows received an intramammary antimicrobial (Dry-Clox; Boehringer Ingelheim Animal Health) and teat sealant (Lockout; Boehringer Ingelheim Animal Health) in all quarters and were housed in the same freestall pen after dry-off. To be eligible for recruitment, cows had to be in good general health, have a BCS >2.0 on a 1 to 5 scale (Ferguson et al., 1994), and have a lameness score ≤2 on a 1 to 5 scale (Sprecher et al., 1997).

Rumination and activity behaviors were recorded continuously using an ear-mounted monitoring system (SCR Heatime Pro+, Allflex Global) that has been validated for monitoring rumination behavior in dairy cows (Schirmann et al., 2009). Data from d −7 to +14 relative to the day of dry-off were extracted from farm monitoring software. Rumination and activity data were extracted as the total minutes per 2-h period. The TDA and TDR were calculated as the sum of the measures over a 24-h period with time of dry-off being the start point.

The parameter used for the sample size calculation was the TDA. It has been previously reported that each 5 kg/d of milk production at dry-off is associated with 19 extra minutes of activity after dry-off (Rajala-Schultz et al., 2018) and that that the administration of acidogenic boluses decreases milk production by 2.5 kg/d (Maynou et al., 2018). Therefore, a minimum of 15 cows per study group was necessary to detect a minimum of 38 min/d difference in TDA when comparing TRT and CON with standard deviation of 36 min/d, with an 80% power at a 5% type-I error risk.

The TDA and TDR in the first 2 wk after dry-off were analyzed using a linear mixed-effects model with repeated measures of day relative to dry-off. Different covariance structures were tested, and an autoregressive covariance structure was ultimately used in the models based on the lowest Akaike information criterion. The fixed effects were study treatment, average milk production during the last week before dry-off, baseline activity or rumination, lactation group (first, second, or ≥third), and interactions between study treatment and time. Baseline activity and rumination were reported as the average (min/d) across the week before dry-off. No adjustment for multiple comparisons was used but TDA and TDR estimates were compared between study groups within each day. The variables used in the final models were baseline activity (for the model analyzing TDA) or baseline rumination (model analyzing TDR), and the interaction between treatment and day. Although offered to the statistical models, lactation group and DIM were not retained in the final models. All statistical analysis was performed using RStudio version 4.1.2.

Cows in both groups had similar characteristics at enrollment (Table 1). Cows in the TRT group were 17 min/d less active than cows in the CON group in the first 2 wk after dry-off (TRT = 415 min/d; 95% CI: 397 to 434 vs. CON = 432 min/d; 95% CI: 415 to 449). The greatest TDA difference between study groups (33 min/d) was observed on the second day after dry-off when TDA was 395 min/d (95% CI: 370 to 420) for the TRT group and 428 min/d (95% CI: 404 to 451) in the CON group. The TDA dynamic in the first 2 wk after dry-off is presented in Figure 1. On average, cows in the TRT group (550 min/d; 95% CI: 539 to 561) and in the CON group (551 min/d.; 95% CI: 541 to 562) had similar TDR during the first 2 wk after dry-off. On the day after the administration of acidogenic boluses, however, cows in the TRT group had a 51 min/d lower TDR (TRT = 437 min/d.; 95% CI: 414 to 461) when compared with the cows in the CON group (CON = 488 min/d.; 95% CI: 466 to 510). The TDR continued to be lower, albeit by a lower margin, in the TRT group for the next 3 d (on average 26 min/d lower). The TDR dynamics by group can be found in Figure 2.

**Table 1:** Descriptive characteristics of the study population at enrollment for cows in the study treatment (TRT; cows receiving 2 acidogenic boluses at dry-off) and control (CON; cows receiving no intervention at dry-off) groups

Item	TRT	CON
Number of cows	30	34
Lactation group, n (%)		
1	11 (36.7)	13 (38.2)
2	9 (30.0)	11 (32.4)
3	10 (33.3)	10 (29.4)
Milk production,^1^, ^2^ kg/d	21.5 (4.7)	20.9 (5.0)
DIM at dry-off^1^	313 (34.6)	313 (38.6)
Days carrying calf (DCC)^1^	218 (11.3)	218 (14.4)
Baseline total daily rumination,^1^, ^3^ min/d	515 (67.8)	503 (56.3)
Baseline total daily activity,^1^, ^4^ min/d	334 (57.5)	351 (75.3)

1 Data presented as mean (SD).

2 Average milk yield in the last week before enrollment.

3 Average of the total daily rumination time from −7 to 0 d relative to dry-off.

4 Average of the total daily activity time from −7 to 0 d relative to dry-off.

**Figure 1:**
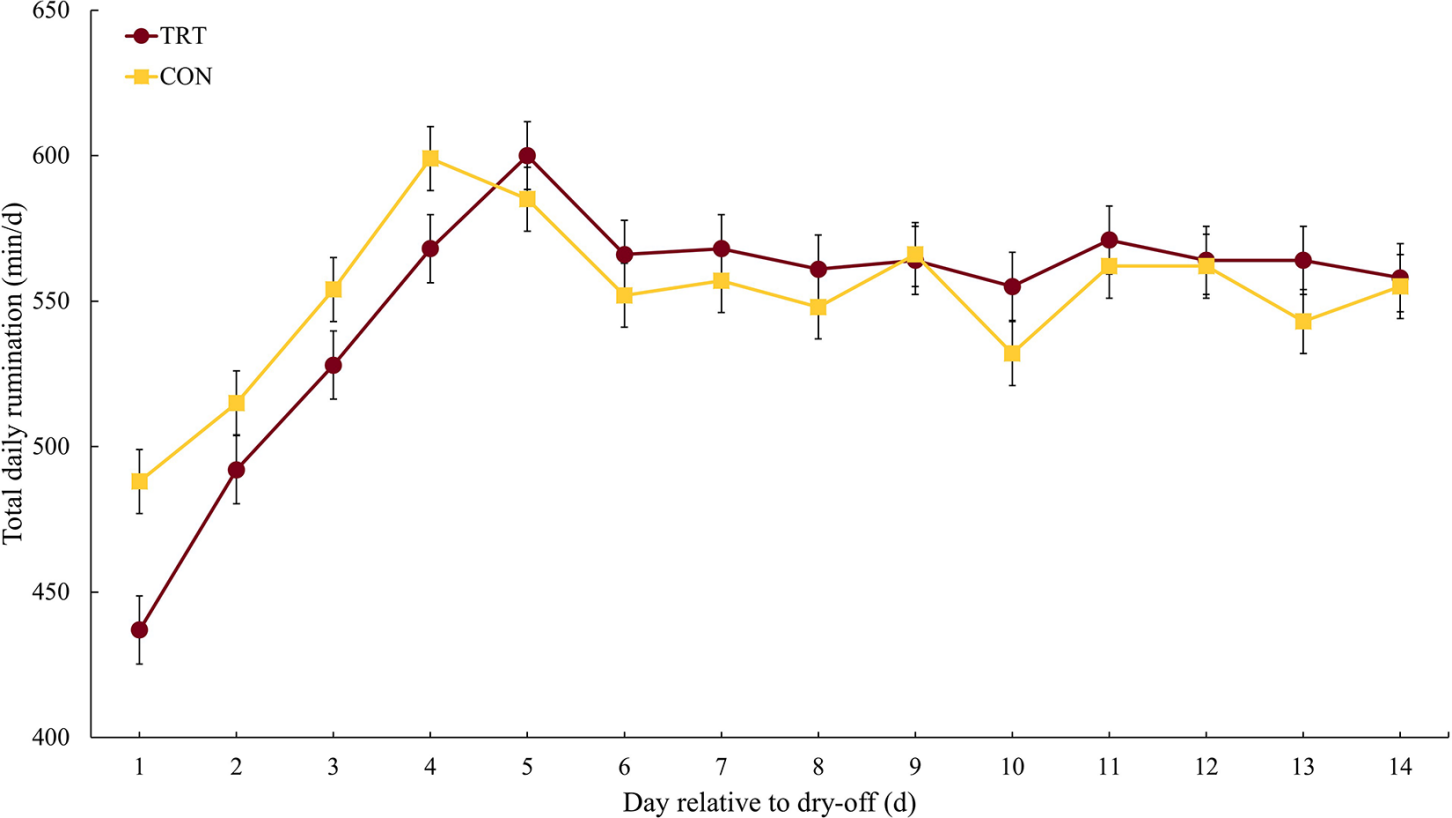
Total daily rumination (TDR) during the first 14 d after dry-off. Acidogenic boluses were administered in the TRT group (n = 30) at dry-off, and TDR was assessed and compared with the control (CON) group (n = 34). Adjusted means are reported in minutes per day for 2 wk during the dry period. The TDR differs between groups in the first day after treatment administration (*P* = 0.002), and it was affected by the interaction of treatment and day (*P* = 0.01), treatment (*P* = 0.001), day (*P* < 0.001), and the baseline for rumination (*P* < 0.001). Error bars represent SEM.

**Figure 2:**
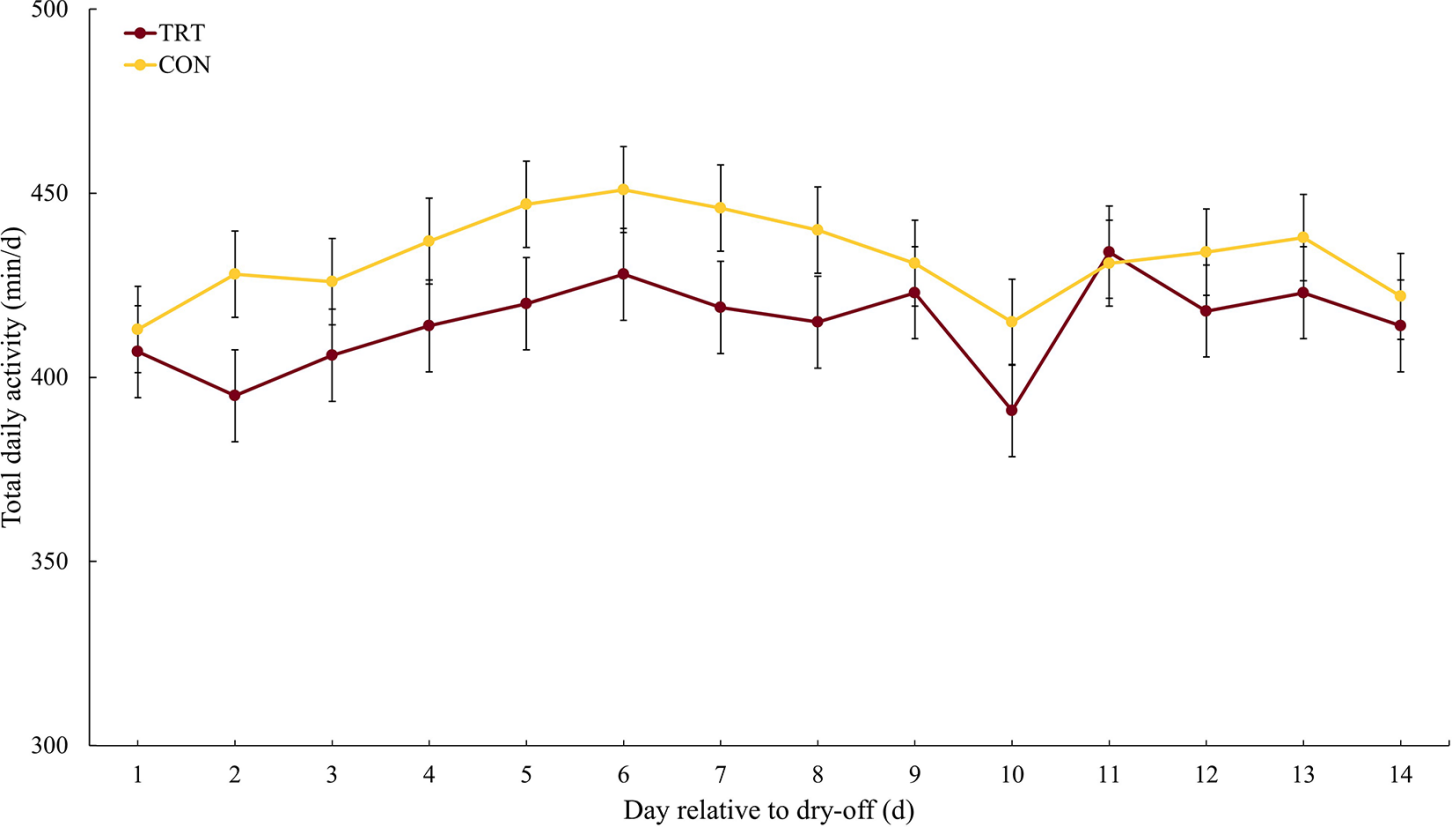
Total daily activity (TDA) during the first 14 d after dry-off. Acidogenic boluses were administered in the TRT group (n = 30) at dry-off, and TDA was assessed and compared with the control (CON) group (n = 34). Adjusted means are reported in minutes per day for 2 wk during the dry period. The TDA did not differ between groups during the follow-up period and was not affected by day (*P* = 0.08); it was also not affected by the interaction of treatment and day (*P* = 0.56), treatment (*P* = 0.75), and baseline for activity (*P* < 0.001). Error bars represent SEM.

This study was conducted to investigate the effect of the administration of acidogenic boluses at dry-off on TDA and TDR in the first 2 wk of the dry period. In our study, cows receiving acidogenic boluses at dry-off (TRT) were less active during the first week of the dry period and had a lower TDR on the day after administration of acidogenic boluses than cows in the CON group.

Activity time for this system is recorded as any time the cow is not lying down. Although no significant differences were found for TDA, cows in the TRT group were 17 min/d less active than cows in the CON group in the first 2 wk after dry-off, and considering how the system measures activity it could be speculated that cows in the TRT group spent more time lying. The greatest difference in TDA (33 min/d) was observed on the second day after bolus administration. This agrees with a previous report showing that the greatest difference in lying time following the administration of acidogenic boluses after dry-off was observed after 24 h (Maynou et al., 2018). Total daily lying time is a well-known and important metric of comfort and welfare in domesticated animals (Munksgaard and Simonsen, 1996). Lying behavior and animal comfort are correlated metrics; however, several external (e.g., housing characteristics, management practices, ability to perform other priority behaviors such as eating) and cow-specific factors (e.g., health status, biological requirements) can influence lying time and lying behavior (Tucker et al., 2021). For instance, a higher activity time during the dry period has been hypothesized to be associated with greater udder pressure exacerbated by external pressure exerted on the udder when lying (Rajala-Schultz et al., 2018). Cows receiving acidogenic boluses, however, have a decreased milk production and reduced udder pressure during the first 2 d after bolus administration (Maynou et al., 2018). Thus, it is reasonable to speculate that the lower activity of TRT cows in our study by comparison to CON cows is a result of cows being more comfortable transitioning into the dry period, likely because of decreased udder pressure, when acidogenic boluses are administered at dry-off.

In our study, cows in the TRT group spent less time ruminating on the first day after dry-off but increased their TDR in the following 3 d to the same levels as cows in the CON group with the same rumination behavior pattern observed in both groups for the remainder of the study period. Adverse health and welfare events lead to changes in different behavioral patterns of dairy cattle, such as resting, feeding, rumination, activity, and even socialization behaviors (Dittrich et al., 2021). Due to its biological importance, changes in rumination have long been associated with impaired health in dairy cows (Radostits et al., 2007). Total rumination time has been used to detect stress (Bristow and Holmes, 2007), distress (Schirmann et al., 2011), clinical diseases (Fogsgaard et al., 2012; Stangaferro et al., 2016a,b), and metabolic disorders (DeVries et al., 2009; Stangaferro et al., 2016c). The administration of acidogenic boluses leads to a mild and temporary metabolic acidosis in dairy cows, as indicated by a decrease in urine pH (Maynou et al., 2018). Therefore, investigating the effect of the administration of acidogenic boluses on rumination behavior has its importance. Although severe metabolic acidosis has been associated with a decrease in rumination time (DeVries et al., 2009), the mild and temporary metabolic acidosis caused by the administration of acidogenic boluses are equivalent to results reported when feeding diets with a negative dietary cation-anion difference, which has no effect on rumination behavior and overall health of dairy cows (Goff et al., 2020). Similarly, in our study, cows in the TRT group had a similar TDR when compared with CON cows a few days after dry-off. The administration of the acidogenic boluses did not lead to long-term impairments in rumination behavior, indicating no negative effects of the intervention.

Conducting a field trial enable us to described TDA and TDR behaviors following the dry-off procedure under commercial conditions. This approach, however, limited our capability to measure other parameters that could have been used to explain some of our findings. Limitations of the trial included a lack of data on milk leakage and udder pressure, as well as DMI after the administration of acidogenic boluses. Milk leakage and udder pressure information can potentially elucidate the hypothesis of decreased milk formation and the idea of increased animal comfort. Rumination behavior during the dry period has been shown to be a poor predictor of DMI (Schirmann et al., 2012). Therefore, incorporating DMI data in future studies is important to understand the effects of this management strategy on feeding behavior. Last, the absence of a placebo group hindered our ability to measure the potential impact of any physical discomfort following the administration of the boluses. It is important to highlight that the differences in TDR and TDA among TRT and CON groups are within the range of normal variation in dairy cows and were smaller than expected when calculating the sample size needed for this study. Thus, a larger study with a greater sample size is warranted to confirm the findings of this study.

The administration of acidogenic boluses at dry-off resulted in a 17 min/d decrease in TDA during the first 2 wk of the dry period with the greatest activity difference observed after 24 h of bolus administration. Moreover, the administration of acidogenic boluses at dry-off led to a decrease in TDR most pronounced within the first 24 h after bolus administration. The results of our study indicate that the administration of acidogenic boluses at dry-off could be used as an alternative management strategy to increase lying time in the first 2 wk after dry-off and facilitate the transition into the dry period.
